# Palladin Compensates for the Arp2/3 Complex and Supports Actin Structures during *Listeria* Infections

**DOI:** 10.1128/mBio.02259-17

**Published:** 2018-04-10

**Authors:** Aaron S. Dhanda, A. Wayne Vogl, Sharifah E. Albraiki, Carol A. Otey, Moriah R. Beck, Julian A. Guttman

**Affiliations:** a Department of Biological Sciences, Faculty of Sciences, Simon Fraser University, Burnaby, BC, Canada; b Department of Cellular and Physiological Sciences, Faculty of Medicine, University of British Columbia, Vancouver, BC, Canada; c Chemistry Department, Wichita State University, Wichita, Kansas, USA; d Department of Cell Biology and Physiology and the Lineberger Comprehensive Cancer Center, University of North Carolina at Chapel Hill, Chapel Hill, North Carolina, USA; University of Washington

**Keywords:** actin nucleation, actin polymerization, Listeria monocytogenes

## Abstract

Palladin is an important component of motile actin-rich structures and nucleates branched actin filament arrays *in vitro*. Here we examine the role of palladin during Listeria monocytogenes infections in order to tease out novel functions of palladin. We show that palladin is co-opted by L. monocytogenes during its cellular entry and intracellular motility. Depletion of palladin resulted in shorter and misshapen comet tails, and when actin- or VASP-binding mutants of palladin were overexpressed in cells, comet tails disintegrated or became thinner. Comet tail thinning resulted in parallel actin bundles within the structures. To determine whether palladin could compensate for the Arp2/3 complex, we overexpressed palladin in cells treated with the Arp2/3 inhibitor CK-666. In treated cells, bacterial motility could be initiated and maintained when levels of palladin were increased. To confirm these findings, we utilized a cell line depleted of multiple Arp2/3 complex subunits. Within these cells, L. monocytogenes failed to generate comet tails. When palladin was overexpressed in this Arp2/3 functionally null cell line, the ability of L. monocytogenes to generate comet tails was restored. Using purified protein components, we demonstrate that L. monocytogenes actin clouds and comet tails can be generated (in a cell-free system) by palladin in the absence of the Arp2/3 complex. Collectively, our results demonstrate that palladin can functionally replace the Arp2/3 complex during bacterial actin-based motility.

## IMPORTANCE

Structures containing branched actin filaments require the Arp2/3 complex. One of the most commonly used systems to study intracellular movement generated by Arp2/3-based actin motility exploits actin-rich comet tails made by *Listeria*. Using these infections together with live imaging and cell-free protein reconstitution experiments, we show that another protein, palladin, can be used in place of Arp2/3 to form actin-rich structures. Additionally, we show that palladin is needed for the structural integrity of comet tails as its depletion or mutation of critical regions causes dramatic changes to comet tail organization. These findings are the first to identify a protein that can functionally replace the Arp2/3 complex and have implications for all actin-based structures thought to exclusively use that complex.

## INTRODUCTION

The infectious cycle of the invasive and motile bacterium Listeria monocytogenes relies heavily on its ability to exploit the epithelial cell actin cytoskeleton. These bacteria require actin filaments for their uptake into epithelial cells (1) and for their formation of motile actin-rich structures generated within their host cells called comet tails (2, 3). During comet tail formation, L. monocytogenes recruits and activates the Arp2/3 complex to one of their poles through the N-WASP-mimicking bacterial protein ActA (4). This recruitment stimulates the force-generating, branched actin polymerization that is needed to propel L. monocytogenes within and among host cells and functions at the molecular level, similarly to lamellipodia in migrating eukaryotic cells (5).

The actin-binding protein palladin is widely expressed in developing and adult vertebrate cells and tissues (6–8). This multifunctional protein acts as a scaffolding protein by recruiting other actin-related proteins, such as profilin (9), VASP (10), α-actinin (11), ezrin (7), PDLIM1 (12), Eps8 (13), and LASP-1 (8), to actin-rich structures within the cell. Palladin can directly cross-link actin filaments, an activity that has been linked to its Ig3 domain (14), and can generate cross-linked actin filaments when pure preparations of palladin and monomeric actin are coincubated *in vitro* (15). In this study, we explore the involvement of palladin during L. monocytogenes infections and simultaneously use L. monocytogenes infections as a model to study the functional roles of palladin.

## RESULTS

### Palladin localizes to L. monocytogenes entry sites and actin-rich comet tails, but not listeriopods.

**FIG 1:**
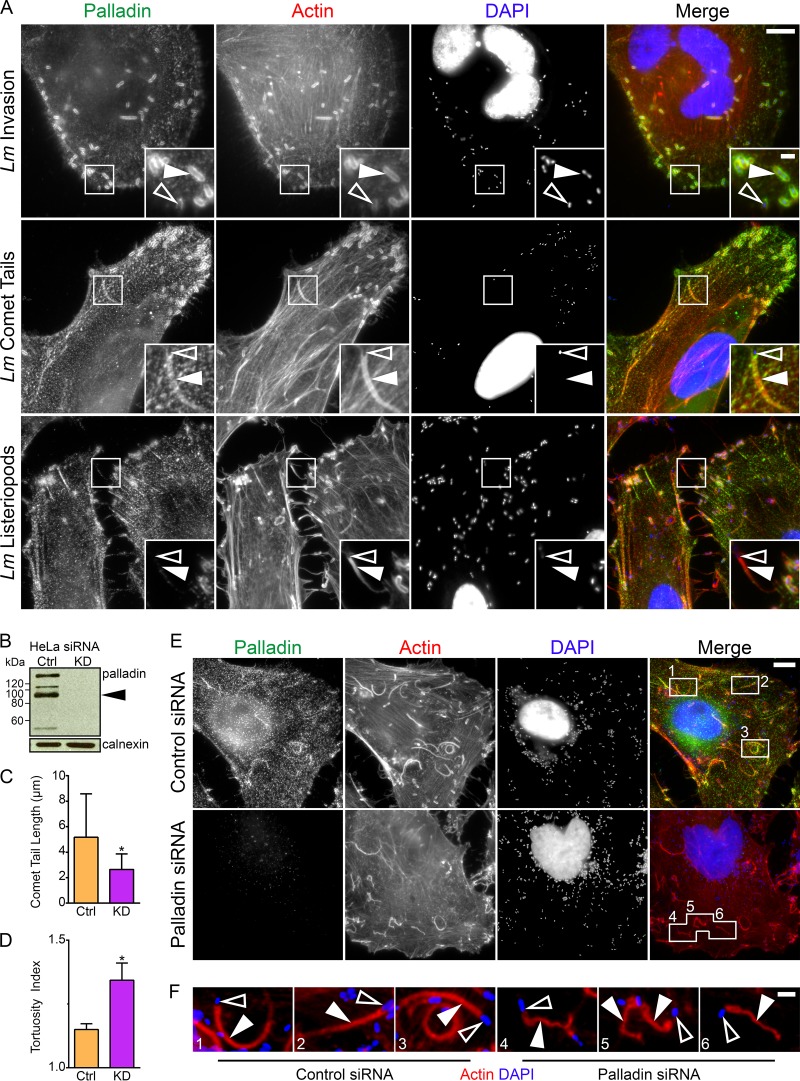
Palladin is hijacked by L. monocytogenes in order to maintain the structural integrity of actin-rich comet tails. (A) HeLa cells were infected with wild-type L. monocytogenes (*Lm*) for 30 min (invasion) or 6 h (comet tail and listeriopod formation), fixed, and stained with a mouse palladin-targeting monoclonal antibody (green), DAPI (blue) to visualize DNA, and Alexa 594-phalloidin (red) to visualize actin. Palladin is recruited to bacterial invasion sites’ actin clouds, and comet tails but not listeriopods (boxed regions). Open arrowheads within insets (enlargement of boxed regions) indicate individual bacteria, while solid arrowheads point to the actin clouds, comet tails, and listeriopods of interest. Scale bars, 10 μm and 2 μm (insets). (B) HeLa cells were treated with nontargeting control (Ctrl) or palladin-targeted (knockdown [KD]) siRNA sequences, and whole-cell lysates were collected and probed for endogenous palladin using a rabbit polyclonal anti-palladin antibody. The black arrowhead indicates palladin isoform 4; additional bands show other palladin isoforms. Calnexin is shown as a loading control. (C and D) Average comet tail length (C) and tortuosity (D) in control (Ctrl) and palladin-targeted (KD) siRNA-treated cells. The average comet tail lengths were as follows: 5.172 μm in control cells and 2.646 μm in knockdown cells. The average comet tail tortuosities (indicated by tortuosity index) were as follows: 1.150 in control cells and 1.343 in knockdown cells. Asterisks indicate *P* < 0.0001 in panel C and *P* < 0.01 in panel D. (E) HeLa cells were treated with nontargeting control or palladin-targeted siRNA sequences and infected with wild type L. monocytogenes for 6 h. Cells were fixed and stained with a mouse anti-palladin monoclonal antibody (green), DAPI (blue) to visualize DNA, and Alexa 594-phalloidin (red) to visualize actin. Comet tails are shorter and severely tortuous in palladin-depleted cells (boxed regions). Scale bar, 5 μm. (F) Enlargement of boxed regions from panel E. Open arrowheads indicate individual bacteria, while solid arrowheads point to comet tails. Note the color levels were modified from panel E to enhance bacteria and comet tails. Scale bar, 1 μm.

L. monocytogenes exploits the actin polymerization machinery at four distinct times: (i) for entry into cells, (ii) as a cloud of F-actin surrounds immotile bacteria, (iii) for intracellular motility, and (iv) for cell-to-cell spreading. When these microbes hijack the actin cytoskeleton, they rearrange it into dynamic morphological structures (actin clouds, comet tails, and listeriopods). To determine if palladin was recruited to these structures, we infected HeLa cells with wild-type L. monocytogenes and stained fixed cells for endogenous palladin. We found that palladin was recruited to bacterial entry sites, actin clouds, and comet tails, but was absent from listeriopods (Fig. 1A). The altered localization of palladin in infected cells did not change its level of expression (see Fig. S1 at https://figshare.com/s/ded44d01de3f33a00800).

Because these bacterially induced actin-based structures are highly dynamic, we sought to determine the recruitment characteristics of palladin in relation to actin. To do this, we cotransfected Ptk2 cells with green fluorescent protein (GFP)-palladin and mKate-LifeAct and saw their simultaneous recruitment to actin clouds surrounding stationary bacteria as well as at motile comet tails (see Movie S1 in the supplemental material). When comet tails pushed motile bacteria into the plasma membrane to initiate listeriopod formation, overall palladin intensity decreased to general cytoplasmic levels, except at regions close to the bacterium-actin interface that continued to show increased palladin recruitment (see Movie S2 in the supplemental material).

10.1128/mBio.02259-17.1 MOVIE S1  Palladin and F-actin colocalize throughout L. monocytogenes motility. mKate-LifeAct (to visualize actin)- and GFP-palladin-cotransfected Ptk2 cells were infected with wild-type L. monocytogenes and visualized 8 h postinfection. GFP-palladin and actin are colocalized at stationary actin clouds and, subsequently, at comet tails during bacterial intracellular motility (arrowheads). Elapsed time is displayed in minutes and seconds. Scale bar, 10 µm. Download MOVIE S1, AVI file, 0.9 MB. Copyright © 2018 Dhanda et al. 2018 Dhanda et al. This content is distributed under the terms of the Creative Commons Attribution 4.0 International license.

10.1128/mBio.02259-17.2 MOVIE S2  Palladin is shed from comet tails during listeriopod formation. Cropped portion of Movie S1 showing GFP-palladin is shed from comet tails during listeriopod formation (arrowheads). Elapsed time is displayed in minutes and seconds. Scale bar, 5 µm. Download MOVIE S2, AVI file, 5.4 MB. Copyright © 2018 Dhanda et al. 2018 Dhanda et al. This content is distributed under the terms of the Creative Commons Attribution 4.0 International license.

### Palladin is required for the proper formation and motility of comet tails.

To investigate the importance of palladin during comet tail formation, we depleted palladin in HeLa cells using small interfering RNA (siRNA) and then infected the cells with L. monocytogenes (Fig. 1B and E). We found no significant effects on bacterial invasion in cells with undetectable levels of palladin compared to control siRNA-treated cells (see Fig. S2A at https://figshare.com/s/f122734e94f779c319fa). Because bacteria could still invade host cells, we examined the morphology of comet tails in palladin-depleted cells and discovered that comet tails were significantly shorter than those in control siRNA-treated cells (Fig. 1C). Actin staining also showed that in palladin-depleted cells, comet tails were distorted compared to their straighter counterparts in control cells (Fig. 1E and F). We quantified this difference by measuring the ratio of the full comet tail length to the shortest distance between the bacterium-tail interface and the end of the comet tail (tortuosity index) and confirmed that comet tails in palladin-depleted cells were severely tortuous (Fig. 1D), further supporting palladin’s role within the eukaryotic cytoskeleton as an important actin-cross-linking protein. Interestingly, listeriopods appeared unaltered in the absence of palladin (see Fig. S2B at https://figshare.com/s/f122734e94f779c319fa).

### Palladin mutations dramatically alter L. monocytogenes motility and F-actin organization within comet tails.

**FIG 2:**
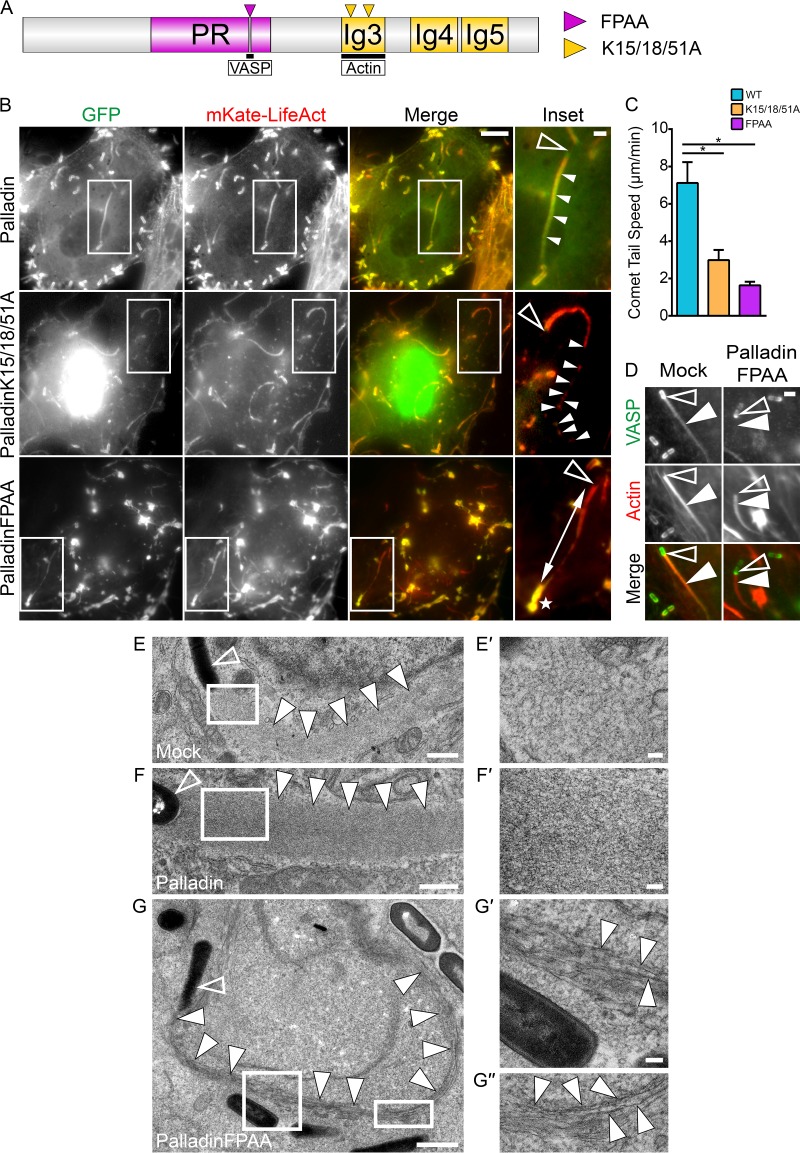
Palladin mutations dramatically alter L. monocytogenes motility rates and F-actin organization within actin-rich comet tails. (A) Schematic diagram of palladin showing the FPAA (purple arrowhead) and K15/18/51A (yellow arrowheads) mutation sites. The proline-rich region (PR) and immunoglobulin-like (Ig) domains are indicated. (B) Still frames from movies of HeLa cells cotransfected with mKate-LifeAct to visualize actin and one of the GFP-tagged wild-type palladin, palladinK15/18/51A, or palladinFPAA DNA constructs and infected with wild-type L. monocytogenes for at least 6 h. Comet tails disintegrate into clumps in GFP-palladinK15/18/51A-expressing cells or become progressively thinner in GFP-palladinFPAA-expressing cells (boxed regions). Open arrowheads within insets (enlargement of boxed regions) indicate individual bacteria, while solid arrowheads point to a normal comet tail (palladin) or broken comet tail clumps (palladinK15/18/51A). The double-headed arrow indicates a thin comet tail (palladinFPAA) tethered to its diffuse actin cloud (white star). Note the inset color levels were modified to enhance comet tails. Scale bars, 5 μm and 1 μm (insets). (C) Mean rates of intracellular movement of wild-type L. monocytogenes. The average bacterial speeds were as follows: 7.117 μm/min in palladin-expressing cells, 2.983 μm/min in palladinK15/18/51A-expressing cells, and 1.635 μm/min in palladinFPAA-expressing cells. *, *P* < 0.01. (D) Endogenous VASP localizes to the bacterial surface and comet tail but is absent from comet tails in palladinFPAA-expressing cells. HeLa cells transfected with palladinFPAA and infected with wild-type L. monocytogenes for 6 h were fixed and stained with a rabbit polyclonal VASP-targeting antibody (green) and Alexa 594-phalloidin (red) to visualize actin. Open arrowheads indicate individual bacteria, while solid arrowheads point to the comet tails of interest. Scale bar, 1 μm. (E to G″) Electron micrographs of wild-type L. monocytogenes comet tails in mock-transfected (E and E′), palladin-transfected (F and F′), and palladinFPAA-transfected (G, G′, and G″) HeLa cells. Higher magnifications in panels E′ and F′ (boxed regions of E and F, respectively) show the highly dendritic actin filament meshwork, whereas comet tails in palladinFPAA-expressing cells consist largely of parallel arrays of actin (G′ and G″). Open arrowheads indicates bacteria, while solid arrowheads point to the comet tails of interest. Scale bars indicate 1 μm in panel G, 500 nm in panels E and F, and 100 nm in insets.

The actin and VASP binding domains of palladin are important for its normal functioning (16, 17). We examined the influence of those regions on L. monocytogenes infections by overexpressing palladin domain mutants that acted in dominant-negative fashions (Fig. 2A).

The actin binding ability of palladin has been tied to three key lysine residues (K15, K18, and K51) within its C-terminally located Ig3 domain (Fig. 2A). When a palladin variant mutated at these residues (palladinK15/18/51A) is expressed in cells, abnormal nuclear localization of palladin and extensive cytoskeletal perturbations in cultured epithelial cells are evident (16). We cotransfected GFP-palladinK15/18/51A (actin-binding mutant) with mKate-LifeAct into HeLa cells and viewed, via time-lapse imaging, motile bacteria during L. monocytogenes infections. We used the known nuclear localization of GFP-palladinK15/18/51A as a readout of cells that had functionally perturbed palladin and found that motile comet tails disintegrated at their distal ends, leaving behind a trail of comet tail debris (Fig. 2B; see Movie S3 in the supplemental material). The crumbling comet tails moved at rates approximately 2-fold slower than comet tails in cells overexpressing GFP-tagged wild-type palladin (Fig. 2C).

10.1128/mBio.02259-17.3 MOVIE S3  L. monocytogenes comet tails disintegrate in palladinK15/18/51A-expressing cells. mKate-LifeAct (to visualize actin)- and GFP-palladinK15/18/51A-cotransfected HeLa cells were infected with wild-type L. monocytogenes and visualized 8 h postinfection. Comet tails (arrowheads) move slower and dramatically disintegrate at their distal ends into clumps (full arrows) during dynamic motility. Elapsed time is displayed in minutes and seconds. Scale bar, 5 µm. Download MOVIE S3, AVI file, 0.8 MB. Copyright © 2018 Dhanda et al. 2018 Dhanda et al. This content is distributed under the terms of the Creative Commons Attribution 4.0 International license.

The actin-associated protein VASP classically localizes to the surface of L. monocytogenes through direct interactions with the bacterial protein ActA; however, it is also present throughout the comet tail (Fig. 2D). To assess the requirement of ActA expression on palladin recruitment to the bacterial surface, we utilized bacteria with *actA* deletion. Not surprisingly, palladin was unable to be redistributed to the surface of these mutant bacteria (see Fig. S3 at https://figshare.com/s/60c87e090bce18cad3ba). Due to the importance of VASP during L. monocytogenes motility (18–21) and its association with palladin at actin-rich structures within the cell (17), we examined the functional role of palladin-VASP interactions during L. monocytogenes infections by using a GFP-tagged palladin construct mutated in its VASP-binding polyproline motif (GFP-palladinFPAA). We found dramatic alterations to the actin cytoskeleton in GFP-palladinFPAA-expressing cells. In these cells, abnormal cytoplasmic clumps and bar-like structures were generated that were enriched with actin and VASP (see Fig. S4A and B at https://figshare.com/s/709e87fc36ce62e52548). We could not identify these unusual structures in either GFP-palladin- or GFP-palladinK15/18/51A-expressing cells (see Fig. S4A). We used these abnormal actin-rich formations together with GFP as convenient markers for cells that were successfully transfected with GFP-palladinFPAA. When actin clouds were examined during live infections, the filamentous actin appeared notably diffuse around nonmotile bacteria (Fig. 2B; see Movie S4 in the supplemental material). Strikingly, when comet tails were viewed, they became progressively thinner as they moved (Fig. 2B; Movie S4). The thin comet tails also remained tethered to remnants of diffuse actin clouds that had stayed behind in their original location surrounding the L. monocytogenes bacteria prior to their launch into dynamic motility (Fig. 2B; Movie S4). We also found that endogenous VASP was redistributed away from comet tails in GFP-palladinFPAA-expressing cells; however, its recruitment to the surface of bacteria remained unaltered (Fig. 2D). Motility rates of bacteria in GFP-palladinFPAA-expressing cells decreased by 4-fold compared to GFP-palladin-overexpressing cells (Fig. 2C).

10.1128/mBio.02259-17.4 MOVIE S4  L. monocytogenes comet tails are thin and tethered to diffuse actin clouds in palladinFPAA-expressing cells. mKate-LifeAct (to visualize actin)- and GFP-palladinFPAA-cotransfected HeLa cells were infected with wild-type L. monocytogenes and visualized 8 h postinfection. Bacteria form diffuse actin clouds (full arrows) that remain behind the bacteria during dynamic motility. Motile comet tails (arrowheads) remain attached to diffuse clouds at their distal ends and move slower, as well as become progressively thinner as they move. Elapsed time is displayed in minutes and seconds. Scale bar, 10 µm. Download MOVIE S4, AVI file, 1.4 MB. Copyright © 2018 Dhanda et al. 2018 Dhanda et al. This content is distributed under the terms of the Creative Commons Attribution 4.0 International license.

To gain further insight into the thinning events occurring at comet tails, we used transmission electron microscopy. Comet tails normally exploit the Arp2/3 complex to generate a branched actin filament network. We found that the arrangement of F-actin throughout the thinned GFP-palladinFPAA comet tails contained parallel arrays of F-actin, whereas mock-transfected and GFP-palladin-transfected cells did not (Fig. 2E to G″). Collectively, these findings show that the actin-binding and scaffolding roles of palladin are crucial for the proper formation and organization of branched actin filament arrays within comet tails.

### Palladin compensates for the Arp2/3 complex during L. monocytogenes actin-based motility.

**FIG 3:**
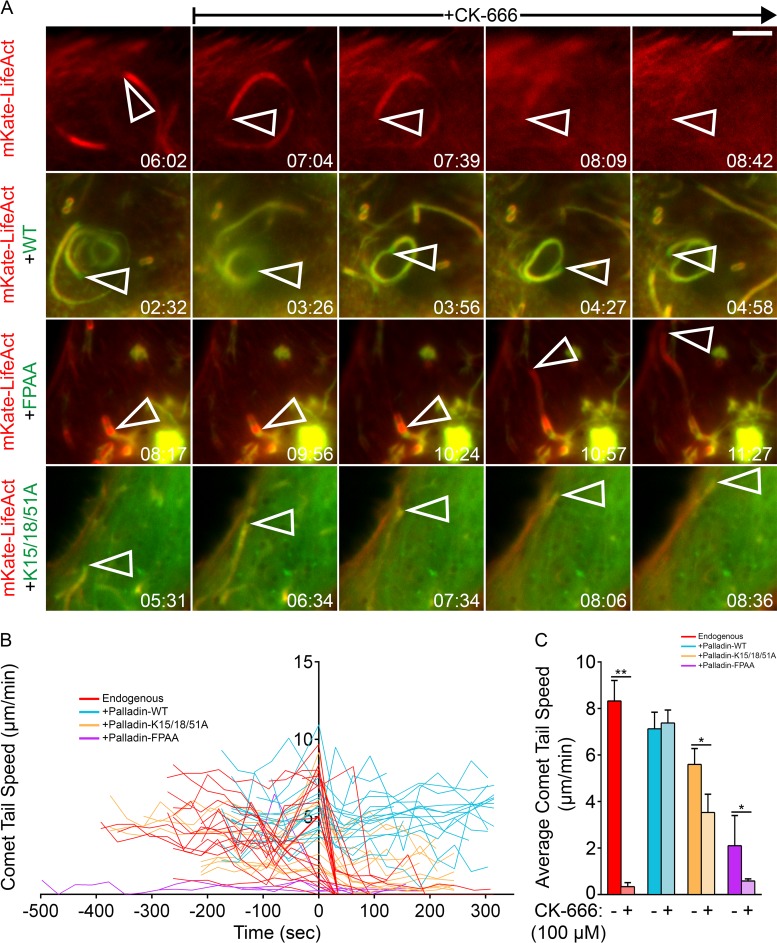
Palladin compensates for Arp2/3 complex functional defects during the intracellular motility of L. monocytogenes. (A) Still frames from movies of HeLa cells transfected with mKate-LifeAct alone to visualize actin or cotransfected with mKate-LifeAct and GFP-tagged wild-type palladin, palladinK15/18/51A, or palladinFPAA constructs and infected with wild-type L. monocytogenes for at least 8 h. Bacterial movement halts immediately and comet tails disintegrate following perfusion of CK-666 (100 μM) in cells with endogenous levels of palladin. Bacterial movement and comet tails are unaffected in cells overexpressing palladin variants in the presence of CK-666 (100 μM). Open arrowheads indicate individual bacteria. Elapsed time is shown in minutes and seconds. Scale bar, 5 μm. (B and C) The effect of CK-666 (100 μM) on the instantaneous (B) and average (C) speeds of L. monocytogenes in cells with endogenous levels of palladin (red) or overexpressing GFP-tagged wild-type palladin (blue), palladinK15/18/51A (yellow), or palladinFPAA (purple) constructs. The origin of the axes indicates the final motility rate measured prior to injection of CK-666 in panel B. Injection of CK-666 significantly decreases bacterial motility rates, except in cells overexpressing wild-type palladin. The mean speed rates (C) of intracellular L. monocytogenes pre-CK-666 injection and post-CK-666 injection are as follows: 8.990 and 0.279 μm/min in mKate-LifeAct-expressing cells, 7.989 and 8.409 μm/min in wild-type palladin-expressing cells, 6.946 and 4.175 μm/min in palladinK15/18/51A-expressing cells, and 1.177 and 0.566 μm/min in palladinFPAA-expressing cells. **, *P* < 0.001; *, *P* < 0.01.

We have recently shown that palladin can generate actin filaments *in vitro* (15). Based on these findings, we investigated whether palladin’s apparent actin-nucleating ability could be exploited by L. monocytogenes to maintain its motility if the Arp2/3 complex was functionally defective. To do this, we examined the motility of L. monocytogenes in cells overexpressing GFP-palladin, GFP-palladinK15/18/51A, or GFP-palladinFPAA prior to (and following) perfusion of the Arp2/3 complex inhibitor CK-666 (22). A cessation of L. monocytogenes motility has already been demonstrated in infected cells treated with CK-666 (22). We confirmed this by live-cell imaging of L. monocytogenes infections of cells with endogenous levels of palladin and noticed an immediate disassembly of actin clouds and comet tails resulting in immotile bacteria when CK-666 was introduced (Fig. 3A; see Movie S5 in the supplemental material). Remarkably, when GFP-palladin was overexpressed in cells, L. monocytogenes comet tails remained motile throughout treatment with CK-666 (Fig. 3A; see Movie S6 in the supplemental material). Additionally, bacteria with actin clouds that were stationary prior to the addition of CK-666 in GFP-palladin-overexpressing cells were still able to generate comet tails and became motile in a manner indistinguishable from bacteria in untreated cells, suggesting that exogenous palladin is not functioning solely as a stabilizer of preexisting comet tails during treatment with CK-666 (see Movie S7 in the supplemental material). Bacterial motility was also evident in cells overexpressing GFP-palladinK15/18/51A or GFP-palladinFPAA (actin- or VASP-binding palladin mutants) after CK-666 was introduced (Fig. 3A; see Movies S8 and S9 in the supplemental material), albeit with the crumbling and thinning comet tail phenotypes described earlier.

10.1128/mBio.02259-17.5 MOVIE S5  L. monocytogenes comet tails and actin clouds disintegrate immediately in the presence of CK-666. mKate-LifeAct (to visualize actin)-transfected HeLa cells were infected with wild-type L. monocytogenes and visualized 8 h postinfection. During imaging, culture medium was replaced with medium containing CK-666 (100 µM; indicated by text at top left), and imaging was allowed to continue. Immediately upon treatment with CK-666, actin clouds (full arrows) disintegrate and motile comet tails halt to a complete stop and disassemble (arrowheads). Elapsed time is displayed in minutes and seconds. Scale bar, 10 µm. Download MOVIE S5, AVI file, 1.5 MB. Copyright © 2018 Dhanda et al. 2018 Dhanda et al. This content is distributed under the terms of the Creative Commons Attribution 4.0 International license.

10.1128/mBio.02259-17.6 MOVIE S6  Palladin overexpression compensates for Arp2/3 complex defects during L. monocytogenes motility. mKate-LifeAct (to visualize actin)- and GFP-palladin-cotransfected HeLa cells were infected with wild-type L. monocytogenes and visualized 8 h postinfection. During imaging, culture medium was replaced with medium containing CK-666 (100 µM; indicated by text at top left), and imaging was allowed to continue. Upon treatment with CK-666, comet tails (arrowheads) continue to move unperturbed by functional inhibition of Arp2/3 complexes throughout the course of drug treatment. Elapsed time is displayed in minutes and seconds. Scale bar, 10 µm. Download MOVIE S6, AVI file, 1.7 MB. Copyright © 2018 Dhanda et al. 2018 Dhanda et al. This content is distributed under the terms of the Creative Commons Attribution 4.0 International license.

10.1128/mBio.02259-17.7 MOVIE S7  Initiation of L. monocytogenes motility is unaffected by CK-666 in cells overexpressing palladin. Shown is a cropped portion of Movie S6 showing L. monocytogenes bacteria (arrowheads) are able to enter dynamic motility similarly to normal untreated cells even when the Arp2/3 complex is functionally inhibited. Elapsed time is displayed in minutes and seconds. Scale bar, 5 µm. Download MOVIE S7, AVI file, 0.3 MB. Copyright © 2018 Dhanda et al. 2018 Dhanda et al. This content is distributed under the terms of the Creative Commons Attribution 4.0 International license.

10.1128/mBio.02259-17.8 MOVIE S8  L. monocytogenes motility is unaffected by CK-666 in palladinK15/18/51A-expressing cells. mKate-LifeAct (to visualize actin)- and GFP-palladinK15/18/51A-cotransfected HeLa cells were infected with wild-type L. monocytogenes and visualized 8 h postinfection. During imaging, culture medium was replaced with medium containing CK-666 (100 µM; indicated by text at top left), and imaging was allowed to continue. Upon treatment with CK-666, comet tails (arrowheads) continue to move unperturbed by functional inhibition of Arp2/3 complexes throughout the course of drug treatment. Elapsed time is displayed in minutes and seconds. Scale bar, 10 µm. Download MOVIE S8, AVI file, 1.5 MB. Copyright © 2018 Dhanda et al. 2018 Dhanda et al. This content is distributed under the terms of the Creative Commons Attribution 4.0 International license.

10.1128/mBio.02259-17.9 MOVIE S9  L. monocytogenes motility is unaffected by CK-666 in palladinFPAA-expressing cells. mKate-LifeAct (to visualize actin)- and GFP-palladinFPAA-cotransfected HeLa cells were infected with wild-type L. monocytogenes and visualized 8 h postinfection. During imaging, culture medium was replaced with medium containing CK-666 (100 µM; indicated by text at top left), and imaging was allowed to continue. Upon treatment with CK-666, comet tails (arrowheads) continue to move unperturbed by functional inhibition of Arp2/3 complexes throughout the course of drug treatment. Elapsed time is displayed in minutes and seconds. Scale bar, 10 µm. Download MOVIE S9, AVI file, 1.2 MB. Copyright © 2018 Dhanda et al. 2018 Dhanda et al. This content is distributed under the terms of the Creative Commons Attribution 4.0 International license.

To gain further insight into this novel function of palladin, we tracked the speed of motile bacteria by time-lapse microscopy (Fig. 3B). Before the addition of CK-666, motile L. monocytogenes bacteria in cells with endogenous amounts of palladin moved at an average speed of 8.990 µm/min (Fig. 3C). Following CK-666 treatment, bacterial speeds dropped significantly to an average of 0.279 µm/min (Fig. 3C). Although the majority of motile bacteria came to a complete stop after the introduction of CK-666, a fraction of the bacteria displayed a slow (1.281 µm/min), jerky movement (Fig. 3B). Interestingly, L. monocytogenes motility rates in CK-666-treated cells overexpressing GFP-tagged wild-type palladin were unaltered upon treatment with CK-666 and displayed speeds similar to those of bacteria in wild-type cells prior to the drug treatment (Fig. 3B and C).

**FIG 4:**
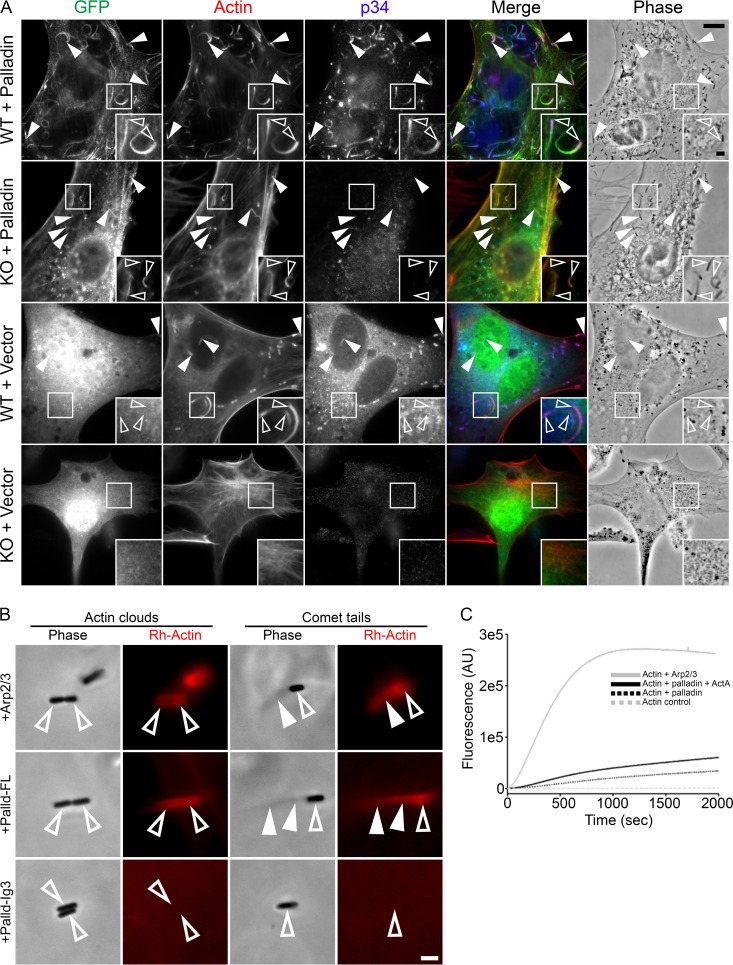
Palladin generates L. monocytogenes actin-rich clouds and comet tails in the absence of the Arp2/3 complex. (A) Wild-type and *Arpc2*^*−/−*^ cells transfected with GFP-palladin or GFP were infected with L. monocytogenes and then fixed and stained with a rabbit polyclonal antibody targeting p34 (blue) to visualize the Arp2/3 complex and Alexa 350-phalloidin (red) to visualize actin. The top row shows wild-type cells containing intracellular bacteria with actin comet tails enriched with palladin and p34. The second row shows cells depleted of the Arp2/3 complex containing intracellular bacteria with actin comet tails enriched with palladin. The third row shows wild-type cells containing intracellular bacteria with actin comet tails enriched with p34 but not GFP. The bottom row shows cells depleted of the Arp2/3 complex containing clustered intracellular bacteria without F-actin at their surface. Open arrowheads within insets (enlargement of boxed regions) indicate individual intracellular bacteria with actin comet tails, while solid arrowheads point to additional intracellular bacteria with actin comet tails. Scale bars, 10 μm and 2 μm (insets). (B) Typical phase-contrast and fluorescent images of actin clouds and comet tails formed in reaction mixtures containing Arp2/3 (top), full-length palladin (middle), or palladin-Ig3 (bottom). Actin clouds and comet tails can be generated by either Arp2/3 or palladin but not the actin-binding domain of palladin. Open arrowheads indicate bacteria, while solid arrowheads point to actin clouds or comet tails. Scale bar, 2 μm. (C) Actin polymerization induced by full-length palladin, Arp2/3, and ActA. Spontaneous assembly reactions were performed by simultaneous addition of actin (5% pyrene labeled, primed with 1 mM EGTA and 0.1 mM MgCl_2_), palladin (10 µM), Arp2/3 (0.37 µM), and ActA (0.3 µM) as indicated. Polymerization was monitored by measuring an increase in fluorescence intensity of pyrene actin in the following reactions: actin alone (gray dashed line), actin with Arp2/3 (gray solid line), actin with palladin (black dashed line), and actin with both palladin and ActA (black solid line).

To further characterize this novel function of palladin, we set out to examine bacterial actin-based motility in cells depleted of the Arp2/3 complex. To do this, we utilized a recently established *Arpc2* conditional knockout cell line (23). In these cells, loss of the *Arpc2* gene product (p34) also caused the depletion of additional Arp2/3 complex subunits, including Arp2 and Arp3 (23). A challenge in studying L. monocytogenes infections in *Arpc2*^*−/−*^ cells was that the bacteria could not invade the cells. To overcome this, we preinfected wild-type cells with L. monocytogenes and then mixed the infected wild-type cells with *Arpc2*^*−/−*^ cells expressing GFP-palladin or the GFP empty vector and allowed the infection to proceed for 16 h, ultimately resulting in the cell-to-cell spread of bacteria into *Arpc2*^*−/−*^ cells. As expected, bacteria within *Arpc2*^*−/−*^ cells expressing GFP alone were unable to generate F-actin at their surface and formed intracellular clusters (Fig. 4A). However, when *Arpc2*^*−/−*^ cells overexpressed GFP-palladin, L. monocytogenes generated actin-rich comet tails enriched with palladin (Fig. 4A), as was the case with wild-type cells expressing GFP-palladin (Fig. 4A).

To definitively establish that palladin can induce F-actin nucleation around bacteria as well as promote the formation and elongation of actin-rich comet tails in the absence of the Arp2/3 complex, we modified a previously developed cell-free protein reconstitution assay (24). This assay allows for the generation of actin clouds and comet tails in a cell-free system when the bacteria are mixed together with purified components of the actin polymerization machinery that include the Arp2/3 complex, profilin, cofilin, gelsolin, and F-actin. As expected, we were able to generate actin clouds and comet tails when the protein constituents contained all of the aforementioned components (Fig. 4B). To test if purified palladin could functionally replace the Arp2/3 complex, we substituted palladin for the Arp2/3 complex. We found that full-length palladin was capable of nucleating F-actin clouds around L. monocytogenes and could polymerize comet tails behind the bacteria (Fig. 4B). We also identified long thin palladin-generated comet tails tethered to clumps of actin (see Fig. S5 at https://figshare.com/s/518f2e2e0875d22da012). None of these structures were generated when the actin-binding domain of palladin (Ig3-palladin) was used in place of full-length palladin (Fig. 4B). When a palladin variant mutated in its VASP-binding region (palladinFPAA) was used in place of the wild-type full-length form, actin clouds and comet tails associated with the bacteria failed to form (see Fig. S6 at https://figshare.com/s/dc0aeaf2a28ebc7f1508). However, this mutant form of palladin did produce two populations of F-actin arrays, linear F-actin bundles and disorganized F-actin clumps (see Fig. S6), that were reminiscent of the structures generated in cells overexpressing palladinFPAA (Fig. 2B).

L. monocytogenes ActA augments the polymerization activity of the Arp2/3 complex (4). Similarly, *in vitro* actin polymerization rates of reaction mixtures containing both Arp2/3 and ActA are enhanced compared to reactions using Arp2/3 alone (4). Using pyrene-actin polymerization assays, we found that ActA also enhanced palladin’s ability to nucleate and polymerize actin (Fig. 4C).

## DISCUSSION

In summary, we have described several novel ways that palladin influences actin-rich structures generated during L. monocytogenes infections. When palladin is depleted from cells, comet tails have a tortuous morphology and a diminished length. When specific amino acids are altered, comet tails crumble and actin organization switches from branched networks to parallel arrays. Moreover, we show that palladin can functionally replace the Arp2/3 complex in infected cells as well as in a cell-free system by promoting the actin-based motility of the bacteria. How does this protein mechanistically accomplish this?

The mutation of three lysine amino acids (from K15, K18, and K51 to A) in the Ig3 domain of palladin that causes crumbling comet tails has previously been shown to severely influence the cross-linking function of the protein, diminishing it to background levels (16). Biochemical evidence has demonstrated that when bound to actin, palladin can also dimerize, which facilitates its cross-linking activity (25). Thus, we postulate that crumbling comet tails may arise due to the diminished ability of palladin to bind actin. In turn, this would lower palladin’s ability to dimerize and thus cross-link actin filaments within the comet tail dendritic meshwork despite the known presence of other potent actin cross-linkers, such as α-actinin and fascin, at the comet tails (26, 27).

Although palladin is crucial for proper comet tail formation, whether its activity is also important during listeriopod formation will require further study. We show that palladin is shed from the actin of the listeriopods, suggesting that it likely has no role in bacterial cell-to-cell spreading events. The altered distribution of actin-associated proteins that are present in comet tails but are eliminated from listeriopods has also been reported for α-actinin (28). Interestingly other proteins work in the reverse manner; being recruited to listeriopods, but not comet tails (28, 29). The differences in actin-associated components are likely due to the morphological organization of the actin filaments within the structures as comet tails contain branched actin arrays and despite Arp2/3 being present at the bacterial actin interface within listeriopods, the majority of actin within those protrusions is organized in a parallel arrangement through formins (28–30).

The role of VASP during L. monocytogenes infections has been studied extensively at the bacterial surface where it directly binds to ActA and regulates L. monocytogenes motility rates (18, 31). However, VASP also localizes within the comet tail, and here we show that an absence of VASP from comet tails in palladinFPAA-expressing cells dramatically influences the organization of actin filaments, changing them from branched to parallel arrays. In addition to the altered organization of F-actin within the comet tails, this mutant form of palladin also dramatically alters the cytoskeleton of the host cell itself as it generates actin-rich cytoplasmic clumps within the cell. Similar clumps were also generated in the cell-free reconstitution assays when palladinFPAA was used in place of wild-type palladin, suggesting that either the lack of the VASP binding domain itself (together with actin) or unknown interactions of the other components in the assays with this palladin mutant are responsible for the observed clumping phenotype. Others have found that the absence of VASP from actin-rich structures increases F-actin branching (32), which may be influencing the observed actin clumps. This would be counterintuitive to our observation of “parallel” structures within the palladinFPAA comet tails. However, in those comet tails VASP remains at the bacterial surface while absent from the length of the comet tail. At the bacterial surface, VASP could function by regulating the elongation of the actin filaments by binding to barbed filament ends associated with the bacterial surface in a process dependent on binding palladin.

Our most significant discovery was the demonstration that palladin can functionally replace the Arp2/3 complex—likely through its ability to nucleate and polymerize F-actin into actin clouds and motile comet tails. We also showed that ActA further enhanced palladin’s actin-nucleating activity, but to a much lesser extent than that generated by the Arp2/3 complex. The substantial disparity between Arp2/3 and palladin plus ActA nucleation/polymerization activities suggests that another mechanism could be involved during the generation of palladin-mediated actin comet tails. In addition to its known actin-nucleating and cross-linking roles, palladin is efficient at stabilizing actin filaments (15). Palladin also dimerizes upon actin binding (25), and this could further enhance both filament cross-linking and stabilization while also providing additional nucleation sites for actin. Thus, as an alternative strategy, palladin may use those properties to enhance its nucleating abilities at L. monocytogenes actin-rich structures.

Because palladin and the Arp2/3 complex are abundant proteins in essentially all epithelial cells, it remains unclear to what extent the replacement of the Arp2/3 complex with palladin would actually occur during *in vivo* infections. Our evidence that palladin itself can nucleate and extend F-actin networks in the complete absence of the Arp2/3 complex is different from what has been shown previously with α-actinin and fascin as these proteins can only extend actin networks generated by the Arp2/3 complex but not nucleate them (27). Because palladin levels are highly elevated at the motile leading edge of normal (10) and wounded cells (33–35), as well as the fact that palladin is highly upregulated in motile cancer cells (36–39), we propose a bona fide cellular function for the actin nucleation role of palladin in enhancing actin-based motility events within the cell. Overall, these findings could have broad implications for an array of cell biology (33, 40), tumor biology (36–39, 41), and microbial pathogenesis (42) fields.

## MATERIALS AND METHODS

### Cell culture.

Human cervical (HeLa) and Potorous tridactylus kidney (Ptk2) epithelial cells were obtained from the American Type Culture Collection (ATCC) (no. CCL-2 and CCL-56, respectively). HeLa cells were cultured in high-glucose Dulbecco’s modified Eagle’s medium (DMEM; Hyclone, GE Healthcare) and were supplemented with 10% fetal bovine serum (FBS; Gibco, Thermo Fisher Scientific). Ptk2 cells were cultured in 1:1 DMEM–F-12 (Hyclone, GE Healthcare) supplemented with 10% FBS. Both cell lines were maintained in a cell culture incubator (37°C, 5% CO_2_). To obtain *Arpc2*^−/−^ cells, the parental mouse embryonic fibroblast (MEF) cell line designated 10-4 was treated with puromycin dihydrochloride (Sigma) and 4-hydroxy-tamoxifen (4-OHT; Sigma), as described previously (23). To seed cells for experiments, flasks or culture dishes with cells were washed 3× with Dulbecco’s phosphate-buffered saline without Ca^2+^ and Mg^2+^ (PBS[−/−]) (Gibco, Thermo Fisher Scientific), trypsinized with 0.05% trypsin-EDTA (Gibco, Thermo Fisher Scientific), and seeded onto clear polystyrene 6-well plates (Corning) containing glass coverslips. For electron microscopy experiments, flexible silicone elastomer membranes (Flexcell International) were used in place of glass coverslips.

### Bacterial strains and growth conditions.

The bacterial strains used were the L. monocytogenes wild type EGD BUG 600 and the Δ*actA* mutant (a gift by Pascale Cossart). All L. monocytogenes strains were grown at 37°C using either brain heart infusion (BHI) agar or broth (BD Biosciences).

### Listeria monocytogenes infections.

To infect cultured cells, broth cultures of L. monocytogenes shaken overnight were diluted 10× in BHI broth and then incubated at 37°C in a shaking incubator until an *A*_600_ of 1.00. Once cultures reached an *A*_600_ of 1.00, bacteria were spun down for 5 min at 10,000 rpm (25°C) and washed with PBS[−/−]. Pelleted bacteria were resuspended with serum-free medium and then diluted 100×. Diluted bacteria were added onto culture plates containing host cells and incubated for 30 min to study bacterial internalization or at least 6 h to study comet tails and listeriopod formation. To infect *Arpc2*^−/−^ cells, wild-type cells were infected for 2 h, collected from culture plates, and overlaid onto culture plates containing uninfected *Arpc2*^−/−^ cells, where the infection was allowed to proceed for an additional 14 to 18 h.

### Antibodies and reagents.

The antibodies used in this study included the following: Alexa Fluor 594-, 488- and 350-conjugated phalloidin from Invitrogen; Alexa Fluor 594-, 488-, and 350-conjugated goat anti-rabbit and goat anti-mouse antibodies (2 µg/ml) from Invitrogen; mouse anti-palladin antibody (50 µg/ml for immunofluorescence and 500 µg/ml for Western blotting) from Novus Biologicals, Inc.; rabbit anti-palladin antibody (0.002 µg/ml for immunofluorescence and 0.002 µg/ml for Western blotting) from the Proteintech Group; rabbit anti-VASP antibody (1.1 µg/ml for immunofluorescence and 0.1 µg/ml for Western blotting) from Sigma; rabbit anti-p34 antibody (10 µg/ml for immunofluorescence) from Millipore; rabbit anti-actin antibody (0.1 µg/ml for Western blotting) from Abcam, Inc.; rabbit anti-calnexin antibody (1:2,000 for Western blotting) from Sigma; and horseradish peroxidase (HRP)-conjugated goat anti-rabbit and goat anti-mouse antibodies (1 µg/ml) from Invitrogen. The reagents used in this study included the following: purified profilin, cofilin, gelsolin, Arp2/3 complex, rhodamine-actin, skeletal actin, and ATP from Cytoskeleton, Inc.; methylcellulose CP4000 and 1,4-diazabicyclo[2.2.2]octane (DABCO) from Sigma; dithiothreitol (DTT) from Sigma-Roche; and CK-666 (100 µM) from Abcam, Inc.

### Immunolocalization.

For immunofluorescence studies, cells were fixed at room temperature for 15 min using prewarmed (37°C) 3% paraformaldehyde (prepared in 150 mM NaCl, 4 mM Na/KPO_4_, 5.0 mM KCl [pH 7.3]) and then washed 3× with PBS[−/−]. Cells were permeabilized with room temperature 0.1% Triton X-100 (prepared in PBS[−/−]) for 5 min and then washed 3× with PBS[−/−]. Samples treated with mouse anti-palladin antibodies required permeabilization with −20°C acetone for 10 min followed by air drying at room temperature for 30 min. All samples were then blocked with 5% normal goat serum (prepared in PBS[−/−]) for 20 min, after which samples were incubated overnight at 4°C with primary antibodies prepared in TPBS/BSA (PBS[−/−], 0.5% Tween 20, 0.1% bovine serum albumin [BSA]). Following this, samples were washed 3× with TPBS/BSA for 10 min and then treated with secondary antibodies (Alexa Fluor 594- or 488-conjugated goat anti-rabbit or goat anti-mouse antibodies) at room temperature for 2 h. To visualize F-actin, samples were incubated with Alexa Fluor 594-, 488-, or 350-conjugated phalloidin (prepared in PBS[−/−]) for 20 min. Samples were washed 3× with PBS[−/−] and mounted onto glass microscope slides using Prolong Diamond antifade mountant containing DAPI (4′,6-diamidino-2-phenylindole; Invitrogen).

### Lysate preparation and Western blotting.

Lysates were prepared by washing the samples 3× with prewarmed PBS[−/−] and then treating the samples with ice-chilled radioimmunoprecipitation assay (RIPA) lysis buffer (150 mM NaCl, 50 mM Tris [pH 7.4], 5 mM EDTA, 1% Nonidet P-40, 1% deoxycholic acid, 10% SDS) with cOmplete Mini EDTA-free protease inhibitor cocktail (Roche) on ice for 5 min. Cells were disrupted using cell scrapers, and lysates were collected into microcentrifuge tubes. Lysates were spun at 4°C and 10,000 × *g* for 10 min to pellet cellular debris; supernatants were collected into fresh microcentrifuge tubes and immediately stored at −80°C. Protein concentrations were determined using a bicinchoninic acid (BCA) assay kit (Pierce). For Western blotting, lysate samples were first prepared in 6× Laemmli buffer and then boiled for 10 min. Equal amounts of protein were loaded onto 10% SDS–polyacrylamide gels and subsequently resolved by electrophoresis. Following separation, gels were rinsed in water for 5 min and then transferred onto nitrocellulose membranes using a Trans-Blot SD semidry transfer cell (Bio-Rad). After the transfer, the membranes were washed for 5 min in TBST (Tris-buffered saline, 0.05% Tween 20), blocked with 4% Blotto (Santa Cruz Biotechnology) prepared in TBST, and then treated with primary antibodies (diluted in TBST plus 1% BSA) overnight at 4°C. Membranes were washed 3× with TBST for 10 min each prior to incubation with secondary antibodies (HRP-conjugated goat anti-rabbit or goat anti-mouse antibodies) for 1 h at room temperature. Membranes were treated with Western Lightning Plus-ECL (PerkinElmer) following the manufacturer’s instructions and visualized using a Fujifilm LAS-4000 imager (Fujifilm). To confirm equal loading, membranes were stripped of bound antibodies with mild stripping buffer (1.5% glycine, 0.1% SDS, 1% Tween 20 [pH 2.2]) and reprobed using antibodies targeting actin or calnexin as outlined above.

### DNA constructs.

All palladin constructs used consisted of the major 90-kDa isoform, commonly referred to as isoform 4. These included GFP vectors containing wild-type palladin and the actin-binding mutant GFP-palladinK15/18/51A. GFP-palladinFPAA, the VASP-binding mutant construct, was generated from the phrGFPIIN-palladin (human isoform 4) construct. For vector controls, the phrGFPIIN vector (Stratagene) was utilized. The phenylalanine and proline residues found in the polyproline region of palladin were both mutated to alanine using site-directed mutagenesis with the following primers: 5′-GTGCCCGACGTGGCCGCACTGCCGCCGCCACCA-3′ and 5′-TGGTGGCGGCGGCAGTGCGGCCACGTCGGGCAC-3′. Plasmids containing mKate-LifeAct or enhanced green fluorescent protein (eGFP)-LifeAct were a gift from Michael Davidson (Addgene plasmid no. 54697 and 54610, respectively).

### Microscopy and live-cell imaging.

Images were acquired using a Leica DMI4000B (Leica Microsystems, Inc.) inverted fluorescence microscope fitted with a Hamamatsu Orca R2 charge-coupled device (CCD) camera (Hamamatsu Photonics). All devices were controlled by MetaMorph Imaging System software (Universal Imaging). The images obtained were evaluated using Metamorph Imaging System software or ImageJ. For live-cell imaging, a Chamlide IC top-stage incubator system was used to maintain a constant temperature and environment of 37°C and 5% humidified CO_2_ gas in air. Bacterial motility rates and comet tails were analyzed using ImageJ. For CK-666 studies, DMEM (without phenol red) plus 10% FBS containing 100 µM CK-666 (prepared in dimethyl sulfoxide [DMSO]) was perfused into magnetic coverslip-containing chambers (Chamlide) during the infections at the indicated times.

### Cell culture transfections.

All DNA transfections of cultured cells were performed using jetPEI or jetPRIME transfection reagents (Polyplus Transfection) and were carried out according to the manufacturer’s instructions. Briefly, cells were transfected and allowed to incubate at 37°C for 4 h. Following this, medium was replaced and cells were incubated for 24 h at 37°C to allow the expression of the respective gene product. Transfected cells were either fixed for viewing by immunofluorescence microscopy or imaged live by time-lapse microscopy.

### Palladin RNAi knockdown.

For RNA interference (RNAi) knockdown, a smart pool (ON-Targetplus siRNA) of 4 palladin siRNAs and 4 nontargeting control siRNAs was purchased from Dharmacon (GE Healthcare). Transfections were performed using the siRNA transfection reagent INTERFERin (Polyplus transfection) following the manufacturer’s instructions. Briefly, 0.1 ml of serum-free medium containing 50 nM siRNA duplexes and INTERFERin was added to cells contained in clear polystyrene 24-well culture plates. To obtain cells with undetectable levels of palladin protein, cells required a 72-h incubation period following the siRNA treatment.

### Electron microscopy.

Medium was replaced with fixative (1.5% paraformaldehyde, 1.5% glutaraldehyde, and 0.1 M sodium cacodylate [pH 7.3] at room temperature) and left for 2 to 3 h, and then the fixative was replaced with buffer (0.1 M sodium cacodylate [pH 7.3], room temperature). The membranes, with attached cells, were gently cut away from cell culture dishes and then further cut into 1- to 2-cm^2^ pieces. The pieces were placed in glass vials and then washed twice (10 min each wash) with fresh buffer. The samples were postfixed for 1 h on ice with 1% osmium tetroxide in 0.1 M sodium cacodylate (pH 7.3). The samples were washed three times (10 min each wash) with ddH_2_O (room temperature) and then stained en bloc with 1% aqueous uranyl acetate (room temperature). The membranes were again washed three times with double-distilled water (ddH_2_O; room temperature) and then dehydrated through an ascending series of ethyl alcohols (30%, 50%, 70%, 95%, 2 × 100% for 10 min each) followed by two treatments with propylene oxide (15 min each). The samples were then placed in 1:1 mixture of propylene oxide and Embed 812 resin (Electron Microscopy Sciences, Hatfield, PA) and left overnight. The following day, the membranes were passed through two changes of 100% Embed 812 resin and then placed on glass slides with the cells faceup. Embedding capsules were filled with resin and inverted onto the membranes, and then the slides with membranes and embedding capsules were placed in an oven (60°C) for the resin to polymerize for 48 h. After polymerization, the capsules (with embedded cells) were carefully separated from the membranes that remained attached to the slides. The cell layers at the surfaces of the block were sectioned en face using a Leica ultramicrotome and collected onto copper grids. The sections were stained with uranyl acetate and lead citrate and then viewed, and images were collected using an FEI Tecnai G2 Spirit electron microscope operated at 120 kV.

### Protein purification.

Full-length 90-kDa human palladin was subcloned into the pTBSG expression vector to allow for expression of a His_6_ fusion protein. The construct was transformed into Escherichia coli BL21(DE3) cells (NEB, Ipswich, MA), and cells were grown at 37°C until the optical density at 600 nm (OD_600_) reached 0.6 in ZYM-5052 autoinduction media. Cell cultures were pelleted down and resuspended in lysis buffer (20 mM sodium phosphate, 300 mM NaCl, 2 mM DTT, 10% glycerol [pH 7.4]), followed by sonication and clearing of cell lysate by centrifugation. Supernatant was incubated with ProteIndex Ni-Penta agarose 6 Fast Flow (Marvelgent Biosciences, Inc., Canton, MA) slurry overnight at 4°C before the protein was purified according to the manufacturer’s guidelines. The elution fraction was dialyzed into S-column buffer A (25 mM KH_2_PO_4_, 50 mM NaCl, 2 mM DTT [pH 6.5]) before being loaded onto a cation-exchange column on an Äkta fast protein liquid chromatography (FPLC) device (GE Healthcare) with a HiPrep SP XL column. The protein was eluted using a gradient with a target of 100% buffer B (25 mM KH_2_PO_4_, 1 M NaCl, 2 mM DTT [pH 6.5]). Fractions containing palladin were confirmed by SDS-PAGE, and protein was dialyzed into storage buffer containing 20 mM HEPES, 150 mM NaCl (pH 7.5), and 2 mM tris(2-carboxyethyl)phosphine (TCEP). Purification of L. monocytogenes ActA-His (strain DPL1545) was performed as previously described (4). The Arp2/3 protein complex was purchased from Cytoskeleton, Inc. Actin was purified from rabbit muscle acetone powder (PelFreez Biologicals) by the method of Spudich and Watt (43) and gel filtered on a 16/60 Sephacryl S-200 column (GE Healthcare Life Sciences). Purified monomeric actin was stored at 4°C in G-buffer (5 mM Tris [pH 8], 0.1 mM CaCl_2_, 0.2 mM DTT, 0.2 mM ATP, 0.02% sodium azide) and used within 2 to 4 weeks. Pyrene-labeled actin was prepared by the reaction of *N*-(1-pyrenyl) iodoacetamide (Sigma-Aldrich) with gel-filtered monomeric actin (G-actin) as described previously (44).

### Reconstitution of *Listeria* actin clouds and comet tails using purified proteins.

On the day of the experiments, fresh stocks of the following were prepared: assay buffer (10 mM HEPES [pH 7.5], 0.1 M KCl, 1 mM MgCl_2_, 0.1 mM CaCl_2_, 1 mM ATP), ATP–1,4-diazabicyclo[2.2.2]octane (DABCO)–DTT (ADD) mixture (made by mixing together 20 µl of 30 mM ATP/60 mM MgCl_2_, 10 µl 2.2 mM DABCO, and 5 µl 0.2 M DTT), 1% methylcellulose CP4000, 10% BSA, and 48 µM Mg–F-actin. To reconstitute actin polymerization around L. monocytogenes (BUG 600), the following reagents were added in order to an Eppendorf tube (at the following final concentrations) to create the reaction mixture: 0.5% BSA, 3.7 µM cofilin, 2.5 µM profilin, 50 nM gelsolin, 75 nM Arp2/3 or 4.875 µM palladin (full-length, Ig3-palladin, or palladinFPAA), 3.0 µl ADD/24-µl reaction mixture, 1 µM rhodamine–G-actin, 2 × 10^8^ L. monocytogenes cells/ml in assay buffer, 7.6 µM Mg–F-actin, and 0.23% methylcellulose. Anywhere from 2 to 5 µl of the reaction mixture was spotted onto a 35-mm Fluorodish (WPI, Inc.) or standard glass microscope slide premarked with a DakoCytomation pen and then covered with a 12-mm glass circle coverslip. Coverslips were added carefully so as to not squish down against the droplet. Samples were then placed onto the microscope to begin phase-contrast as well as fluorescence microscopy observation of actin cloud and comet tail formation.

### Pyrene fluorescence assay.

Pyrenyl actin and unlabeled G-actin were mixed together to make 5 μM 5% pyrene-labeled G-actin stock. Labeled G-actin (100 μl) was incubated with (10 μl) of 10× priming solution (10 mM EGTA, 1 mM MgCl_2_) for 2 min, and then polymerization was induced by adding 0.37 μM Arp2/3 and 10 μM full-length palladin with or without 0.3 μM ActA. All reactions were performed in G-buffer (without KCl). Pyrene fluorescence was measured immediately with excitation at 365 nm and emission at 385 nm on a PTI fluorescence spectrometer.

### Statistical analysis.

Statistical analysis was performed with Student’s unpaired *t* test (Mann-Whitney *U* test for nonparametric tests) or 1-way analysis of variance (ANOVA) with the Tukey *post hoc* test when comparing means of two or more groups in GraphPad Prism version 6.01. All data are presented as bar graphs with mean ± standard error of the mean (SEM) unless indicated otherwise. Experiments were performed at least 3 times (*n =* 3), and the person who performed the experiment performed all of the enumeration and statistical analyses. All data involving immunofluorescence microscopy were obtained from experiments performed at least 3 times (*n =* 3). The presented microscopy images are representative of experiments performed. In Fig. 1C, at least 45 comet tails were measured per condition per experiment (230 and 162 comet tails in total were measured in control and knockdown cells, respectively). In Fig. 1D, at least 45 comet tails were measured per condition per experiment (116 and 110 comet tails in total were measured in control and knockdown cells, respectively). Data in Fig. 1C depict average values (±standard deviation) from 3 independent experiments, with the 99% confidence interval indicated by an asterisk. Data in Fig. 1D depict average values (±standard error) from 3 independent experiments, with the 99% confidence interval indicated by an asterisk. In Fig. 2C, data show average values (±standard error) from at least 10 rate measurements, which were performed per group over the course of 3 independent experiments, with 99% confidence intervals indicated by asterisks. For Fig. 3B, the instantaneous speeds of 19, 16, 13, and 4 bacteria in cells expressing mKate-LifeAct, mKate-LifeAct and GFP-palladin, mKate-LifeAct and GFP-palladinK15/18/51A, or mKate-LifeAct and GFP-palladinFPAA, respectively, were measured with the total time of bacteria being measured ranging from a minimum of approximately 5 min up to approximately 10 min. For Fig. 3C, data show average values (±standard error), with 90% and 99% confidence intervals indicated by asterisks.
